# Unlocking the Potential of Lignocellulosic Biomass Dragon Fruit (*Hylocereus polyrhizus*) in Bioplastics, Biocomposites and Various Commercial Applications

**DOI:** 10.3390/polym15122654

**Published:** 2023-06-12

**Authors:** N. H. Taharuddin, R. Jumaidin, M. R. Mansor, K. Z. Hazrati, J. Tarique, M. R. M. Asyraf, M. R. Razman

**Affiliations:** 1 Fakulti Kejuruteraan Mekanikal, Universiti Teknikal Malaysia Melaka, Hang Tuah Jaya, Durian Tunggal 76100, Malaysia; hanantaharuddin@gmail.com (N.H.T.); muhd.ridzuan@utem.edu.my (M.R.M.); 2 German-Malaysian Institute, Jalan Ilmiah Taman Universiti, Kajang 43000, Malaysia; hazrati88@gmail.com; 3 Fakulti Teknologi Kejuruteraan Mekanikal dan Pembuatan, Universiti Teknikal Malaysia Melaka, Hang Tuah Jaya, Durian Tunggal 76100, Malaysia; 4 Institute of Energy Infrastructure, Universiti Tenaga Nasional, Jalan IKRAM-UNITEN, Kajang 43000, Malaysia; tarique.jamal@uniten.edu.my; 5 Engineering Design Research Group (EDRG), Faculty of Mechanical Engineering, Universiti Teknologi Malaysia, Johor Bahru 81310, Malaysia; 6 Centre for Advanced Composite Materials (CACM), Universiti Teknologi Malaysia, Johor Bahru 81310, Malaysia; 7 Research Centre for Sustainability Science and Governance (SGK), Institute for Environment and Development (LESTARI), Universiti Kebangsaan Malaysia (UKM), Bangi 43600, Malaysia

**Keywords:** *Hylocereus polyrhizus*, dragon fruit, peel, pectin, natural fibre, pitaya

## Abstract

Dragon fruit, also called pitaya or pitahaya, is in the family Cactaceae. It is found in two genera: ‘*Selenicereus*’ and ‘*Hylocereus*’. The substantial growth in demand intensifies dragon fruit processing operations, and waste materials such as peels and seeds are generated in more significant quantities. The transformation of waste materials into value-added components needs greater focus since managing food waste is an important environmental concern. Two well-known varieties of dragon fruit are pitaya (*Stenocereus*) and pitahaya (*Hylocereus*), which are different in their sour and sweet tastes. The flesh of the dragon fruit constitutes about two-thirds (~65%) of the fruit, and the peel is approximately one-third (~22%). Dragon fruit peel is believed to be rich in pectin and dietary fibre. In this regard, extracting pectin from dragon fruit peel can be an innovative technology that minimises waste disposal and adds value to the peel. Dragon fruit are currently used in several applications, such as bioplastics, natural dyes and cosmetics. Further research is recommended for diverging its development in various areas and maturing the innovation of its usage.

## 1. Introduction

Dragon fruit, also known as pitaya or pitahaya, is a member of the Cactaceae family and is found in two distinct genera: ‘*Selenicereus*’ and ‘*Hylocereus*’. Dragon fruit come from the vine cactus species, which belong to genera *Hylocereus* (Berger) Britton and Rose (Cactaceae); they are native to Mexico, Central America and South America [1]. The *Hylocereus* includes around 16 distinct species and contains the most commercially grown plants [2]. Dragon fruit, also known as “buah naga” or “buah mata naga” in Malaysia, were initially introduced in 1999 in the states of Setiawan, Johor, and Kuala Pilah [3]. The annual production in Malaysia is estimated to be about 10,961 tons [4].

A strawberry pear, *thang loy* (Vietnamese), *pitayaroja* (Spanish), and *la pitahaya rouge* (French) are different names for dragon fruit. On a 6 m long ascending cactus, the fruits are cultivated. This plant grows better in regions with lower yearly rainfall [2]. Dragon fruit plants usually climb and attach themselves to natural or artificial support in vertical or horizontal directions. Dragon fruit plants are semi-epiphytic plants that prefer dry, tropical or subtropical climates with an average temperature of 21–29 °C. However, they can withstand temperatures of 38–40 °C and freezing temperatures (as low as 0 °C) for short periods [5]. The plant is generally grown with vertical supports made from wood or cement, and each stem must be attached to support with a clip.

The main advantage of producing dragon fruit plants is that they mature and survive for around 20 years after planting. About 800 plants can be accommodated in a hectare. More importantly, because it is a perennial crop with a quick return, production starts the year after planting and can be completed in five years [2].

Pitaya species typically occur in Mesoamerica, in terrains varying from a few to 1700 metres above sea level, with 500 to 2000 mm of rainfall [6]. About 20 countries, such as Thailand, Indonesia, Taiwan, Vietnam, Sri Lanka, Bangladesh, Japan, Malaysia, the Philippines, Australia, the United States, and China, have many growing areas. India buys dragon fruit from other countries but has expanded its farms [2].

Most of the time, dragon fruit are eaten raw or are turned into juice. Most dragon fruit have between 36.70 and 37.60% peel, usually thrown away during the process, especially in the drinking industry. This may be harmful to the climate [2,7]. This view is supported by Chia and Chong [8], who mentioned that dragon fruit peel is an agricultural waste in fruit juice processing industries and is currently only used as fertiliser. 

According to Mahlil et al. [9], 1 Ha of dragon fruit plantations will produce 50 tons of fresh dragon fruit, and they anticipated that the same plantation could have as much as 11 tons of dragon fruit peels (calculated as 22% from the 50 tons of fresh dragon fruit). Furthermore, Vietnam, the leading producer of dragon fruit, produces around 1 million metric tons annually, and is estimated to discard around 220,000 tons of dragon fruit peels. This estimation concludes that dragon fruit peel is abundantly available and can be potentially used in bioproducts. 

As a potential tropical fruit that may be grown in various tropical and subtropical parts of the globe, including Southeast Asia, Central America, and South America, Liaotrakoon [5] analysed the trend in dragon fruit (*Hylocereus* spp.) planting. Because of the rapidly growing demand, dragon fruit are now available in practically all exotic fruit stores throughout the globe. This success can be attributed partly to the fruit’s attributes (such as appearance, nutritional content, and health benefits) and to commercial strategies of countries that produce and export dragon fruit, such as Thailand and Vietnam, which are the primary fruit producers.

Thus far, several studies have indicated that dragon fruit and its products may be used as ingredients for innovative foods that respond well to consumer interests [5,10,11]. Liaotrakoon [5] adopted a more critical perspective and said that a significant rise in dragon fruit processing has led to considerable waste production, including peels and seeds. Converting wasted food into value-added components is essential for environmental sustainability since managing food waste is a severe environmental problem. The dragon fruit quality and by-products can be improved by advancing knowledge about its characterisation [5].

## 2. Physiology and Morphology

The peels (36.70–37.60%), pulps (47.40–73.76%), and seeds (2.70–14.67%) are the three main components of dragon fruit [12,13]. Fruit pulps may range from white to shades of purple and red. The dragon fruit peel has either a red or yellow colour. The peel of the red dragon fruit has a deeper shade of red colour than the peel of the white dragon fruit. The seeds are edible, tiny in size, soft, and black in colour [2]. 

The most commonly grown varieties of dragon fruit are white dragon fruit, red dragon fruit and yellow dragon fruit. As for white and yellow dragon fruit, they possess an oblong fruit shape along with white pulp colour; meanwhile, red dragon fruit is commonly found in a rounded fruit shape with a red–violet pulp colour. On the other hand, the peel colour for both white and red dragon fruit is red, while yellow dragon fruit have yellow-coloured skin. 

According to Bakar et al. [7], fresh dragon fruit peel contains 92.65% water, 0.95% protein, 0.10% lipid, 0.10% ash, 6.20% carbohydrates, 150.46 mg/100 g betacyanin, 10.8% pectin and up to 69.30% of the total dietary fibre. That is to say, the main composition of the peel, other than moisture, is fibre. Based on the dry weight, the red dragon fruit peel powder contains 9.47% water, 16.2% ash, 8.9% crude protein, 3.18% lipid, 24% crude fibre, 0.68% Ca, 0.84% available P and EM 2031 Kcal g^−1^, 8.84% lignin, 699.14 mg/100 g tannin, 108.55 mg/100 g anthocyanin, 3.033 ppm lycopene and 5.569 ppm β-carotene. A pectin source is indicated in the dragon fruit pulp [14] and even in the dragon fruit peel [7].

Every part of the dragon fruit, including the pulp, peel, seed, flower bud, and dried flowers, is an excellent source of antioxidants, protein, vitamin C, and minerals, mainly calcium and phosphorus. The fruit has received much attention from researchers due to its nutritional characteristics. Table 1 presents the different products made from processed dragon fruit.

## 3. Varieties 

As previously mentioned, two well-known varieties of dragon fruit are pitaya (*Stenocereus*) and pitahaya (*Hylocereus*). The varieties are distinguished by their tastes, which are sour and sweet, respectively. 

### 3.1. Pitaya (Stenocereus)

Pitaya (*Stenocereus* spp.) is an essential crop for communities of Mexico’s semiarid zones, owing to its nutritional, medicinal, and ornamental properties [15]. *Stenocereus* are spreading plants that grow in groups and look like trees. *Stenocereus* cacti have stems that are round and that often have many ribs. This plant has flowers resembling funnels, which is common among Cactaceae. At least 24 species of *Stenocereus* can be found from the southern United States to Peru and Venezuela. Mexico is home to at least 19 different species [15].

Pitaya is commonly referred to locally through many different and colourful common names, such as amarilla (yellow), hormiga (ant), cantaro (pitcher), acateca, borrega (sheep), espina negra (black spine), agria (sour) or espina amarilla (yellow spine), copal, pitaya senora (mistress pitaya), pitaya de flor o chica (flower pitaya or small), pitaya xoconostle and cantarito (little pitcher) [15].

It has been shown that *Stenocereus* develops well in sandy, somewhat acidic soil, yet it can also thrive in rocky soil where no other plants can. It grows best at temperatures between 10 and 34 °C, with a mean temperature of 22 °C in the Sayula Basin [15]. Between the first and third year after planting, the plant starts to produce flowers and fruit, but it takes 10 years for the plant to reach a sizeable output that is commercially viable.

Pitaya is not extremely prolific compared to other fruits. The main output of fruit globally is 27 mt ha^−1^, whereas the production of prickly pears, a related cactus fruit, is 7.23 mt ha^−1^. Yield per hectare is, on average, just 3.44 metric tonnes (mt). 

#### Physicochemical Characteristics

The pulp (in its internal chambers), seed (found inside pulp), and high-fibre peel are three primary anatomical elements of the pitaya fruit, as described by Chuck-Hernández, Parra-Saldívar and Sandate-Flores [15]. The pulp is fragrant, juicy, and sugary. The pulp may be any of many colours, including pink, white, yellow, purple and red.

The weight of the fruit changes remarkably; in ready pitaya, it might be anywhere between 69.3 and 148.3 g, of which the pulp ranges from 53.9 to 111.8 g, the skin ranges from 15.3 to 36.5 g, and the seeds range from 1.63 to 3.42 g. The pitaya peel accounts for 18% to 24% of the fruit’s weight. Pitaya is approximately spherical in shape, having transverse and longitudinal dimensions of 4.4–6.2 and 4.4–6.8 cm, respectively.

The chemical characteristics of pitaya’s fifteen species or varieties are shown in Table 2. Acidity and pH are linked qualities, with the former ranging from 0.09 to 0.64 and the latter from 3.90 to 5.20. In terms of these characteristics, specific pitaya cultivars are low in acidity since their pH is more than 4.5, necessitating careful attention throughout processing, particularly in thermal-related processes such as pasteurisation.

Pitaya fruit are highly valued in exotic fruit markets, but the short shelf life makes postharvest treatments difficult. In an attempt to help regional farmers, suggestions are made for the industrial and consumer use of pulps and creative ways to use pitaya fruit peel or leftovers in new products [15]. 

### 3.2. Pitahaya (Hylocereus)

Pitahaya is a member of the Cactaceae plant family’s *Hylocereus genus*. The climbing plant *Hylocereus* is distinguished by its aerial roots and glabrous fruit with numerous scales on its peel [10]. The generic term ‘pitahaya’ includes sixteen different species. However, currently, only a few species of pitahaya are usually found on the market. There are three varieties: *Selenicereus megalanthus* (yellow dragon fruit), *Hylocereus undatus* (white dragon fruit), and *Hylocereus polyrhizus* (red dragon fruit) [2]. 

Most *Hylocereus* species are mainly from Latin America (likely from Mexico and Colombia), while some may also come from the West Indies. The *Hylocereus* species may thrive in various ecological settings because of their hardiness. For instance, in Mexico, they may be found up to 2750 m above sea level and in areas that receive 340–3500 mm of rain annually [10]. Pitahaya is grown on divided, vine-like crawling cacti that perform well in semiarid regions but that need a warm environment with increased rainfall. Typically, the fruit requires temperatures of 38–40 °C and rainfall of 600–1300 mm (24–52 in) [16].

#### Physicochemical Characteristics

Fresh pitahaya has interesting nutritional and functional components. According to Bakar et al. [7], the flesh constitutes about two-thirds (~65%) of the fruit, and the peel comprises approximately one-third (~22%). One interesting finding is that the moisture content of the peels was around 93% (wet basis), which was higher than the one told by M et al. [17]. Makris, Boskou and Andrikopoulos [18] and Chantaro, Devahastin and Chiewchan [19] reported that the water contents of the obtained pitaya peels are almost comparable to the water content of other fruit peels such as potato (81.83%), carrot (91.05%), red apple (81.68%) and white grapes (75.28%).

Table 3 lists the physicochemical properties of the peel. The pH of the peel is about 5, which is low in both titratable acidity and total soluble solids. According to Grigelmo-Miguel and Martn-Belloso [20], the soluble dietary fibre (SDF) and insoluble dietary fibre (IDF) of pitaya peels are higher than those of other fruits or plants such as pears (14.1% SDF, 22% IDF), oranges (13.6% SDF, 24.2% IDF), peaches (9.7% SDF, 26.1% IDF), artichoke (14.3% SDF, 44.5% IDF) and asparagus (10.4% SDF, 38.6% IDF). In food, a 3:1 ratio of IDF to SDF is advised. 

## 4. Surface Treatments

In order to acquire the most significant amount of cellulose with greater degrees of crystallinity, surface treatments are often performed for composites reinforced with natural fibres to eliminate amorphous elements such as hemicellulose and lignin. A greater cellulose concentration in the fibre is linked to better mechanical characteristics and thermal degradation resistance [21]. Three methods of natural fibre surface modifications are categorised as biological, chemical, and physical. 

### 4.1. Chemical Treatment

Natural fibre offers the benefits of being cheap, biodegradable, and low density. However, poor connections between the fibre and matrices and relatively high moisture absorption are the main drawbacks of natural fibres in the composite. Consequently, to change the surface characteristics of the fibre, chemical treatments are required [22]. According to Jabbar [23], these methods decrease the hydrophilic trend of natural fibres, enhancing the compatibility of natural fibres with the matrix.

Li, Tabil and Panigrahi [22] identified some substances that improve adhesion by chemically coupling adhesives to the materials, including sodium hydroxide, malleated coupling agents, acrylic acids, silane, acetic acid, isocyanate, peroxide, potassium permanganate.

Chemical coupling substances are compounds that have two functions: they react with cellulose hydroxyl groups and matrix functional groups. Bledzki and Gassan [24] described several mechanisms of coupling in materials, including (a) the removal of weak boundary layers; (b) the formation of the complex and flexible layers; (c) the formation of an interphase zone that is heavily cross-linked and has a modulus that is halfway between that of the substrate and the polymer; (d) improved wetting between polymers and substrates; (e) the development of covalent bonds with both materials; and (f) the modification of the acidity of the substrate’s surface [22].

Several new studies investigating the effect of modifications on dragon fruit peel have been conducted using the suggested chemical compound. A procedure modified by Koubala et al. [25] was used by Ismail et al. [3] in their study on the treatment of dragon fruit peel. The dried peel was treated four times using isopropanol (85 vol.%) at 70 °C for 20 min to produce an alcohol-insoluble residue (AIR). Employing ammonium oxalate (0.25%), pH 4.6 ± 0.01 (adjusted with oxalic acid) at 85 °C, hydrochloric acid (0.03 M; pH 1.49 ± 0.02) at 85 °C, and deionised water at 75 °C, three extraction condition were used and examined to evaluate the properties of dragon fruit pectins. The temperature for all extractions was kept constant, which was 1 h.

The extracted dragon fruit pectin yields from the dried peels varied from 14.96 to 20.14%. The highest results obtained from ammonium oxalate/oxalic acid extractions are feasible for commercial use. The moisture content for all samples was reasonably high (11.13–11.33%) and did not show any significant difference (*p* < 0.05). Mohamadzadeh et al. [26] highlighted the relevance of low moisture content in maintaining the pectin’s quality. Ammonium oxalate-extracted pectins also had reduced ash contents compared to hydrochloric acid and water-extracted pectin, which was 6.88 ± 0.42% and following the criteria of good-quality gel (less than 10%). Dragon fruit pectins had a lower methoxyl concentration and a higher esterification degree than commercial apple pectin. Because pectin made from dragon fruit in this research contains less than 50% DE and a methoxyl content ranging from 0.5 to 7.0%, it can be categorised as minimal methoxyl pectin (LMP) [3].

Similarly, in another research work by Mahlil et al. [9], dragon fruit peels were soaked in acetic acid for different intervals (0, 2, 4, 6 and 8 h), and each treatment was repeated four times. The results indicated that absorbing dragon fruit peel in 7.5% acetic acid for various durations was highly significant in reducing crude fibre contents (*p* < 0.01) in dragon fruit peel but not the crude protein, which can be utilised as alternative poultry.

### 4.2. Physical Treatment

Physical processes can modify natural fibres, including corona, plasma, and steam explosion as well as high-energy irradiations such as laser, UV, and gamma rays. These approaches are mainly used to enhance fibre/matrix adhesion by modifying the structural and surface characteristics of the fibre. All physical modifications are deemed to be environmentally friendly.

Mahlil et al. [27] examined the trend of anthocyanin contents in dragon fruit peels before and after treatment through chemicals as well as physical, biological and physical–biological methods. The conducted physical modification (steaming at 98 °C for 20 min) showed an increase in anthocyanin contents of the dragon fruit peels, from 198.87 to 353.20 ppm. Similarly, Mulyawanti, Budijanto and Yasni [28] discovered that the anthocyanin content of purple sweet potatoes somewhat increased. Due to the -(1,4)-glycoside bond being broken down in the crude fibre fraction of the peel’s cells by steaming, the physical technique of processing dragon fruit peel produced the peel with the greatest anthocyanin concentration. When maceration of the dragon fruit peel was completed, the anthocyanin was readily released from the cells and dissolved in a solution (potassium chloride and natrium acetate) [27].

### 4.3. Biological Treatment

Biological treatment offers an environmentally friendly method for surface modification of natural fibre over chemical and physical treatments. It can selectively remove pectin and hemicellulose, requiring less energy input [29]. Due to this fact, this method is rapidly evolving. The biological agents consist of enzymes, bacterial cellulose and fungi.

To better understand the mechanism of enzyme treatments and their effects, Kunnika and Pranee [30] analysed the quantity of bioactive compounds, colour stabilities of dragon fruit flesh, and peels after treatment. The pre-treatment conditions of heating at 85 °C for three minutes and adding ascorbic acid concentrations of 0.2 and 0.1% (*w*/*w*) were conducted to inhibit browning reactions in the flesh and peels, respectively. Samples were deteriorated by commercial pectinase enzymes generated from *Aspergillus aculeatus* with 10,292 PGU/mL enzyme activity. Enzyme reactions were stopped by heating at 95 ± 50 °C for five minutes.

The results showed that the longer degradation time and higher concentration had an impact on the polysaccharide breakdown rate in both the meat and peel by producing noticeably more lowering sugar (RS) (*p* ≤ 0.05), from 25.61 to 70.56 in the flesh and from 20.35 to 44.54 mg glucose/g FM in the peels. It can be concluded that the dragon fruit flesh has more bioactive compounds than the peel, except for the soluble fibre content [30].

## 5. Dragon Fruit Extraction/Processing 

### 5.1. Pectin Extraction

Pectin is a colloidal, amorphous, white substance with a high molecular weight. It is present in mature fruits, particularly apples, currants, and other similar fruits. Because of its thickening and emulsifying abilities and its gel capacity, it is commonly used in fruit jellies, medications, and cosmetics. These properties and applications have placed pectins in the biopolymer market, possessing great potential and possibilities for development [31]. 

Pectic substances have been identified in numerous plant species and fruits’ primary cell walls and middle lamellae. These entities are commonly linked with the structures of cellulose, hemicellulose, and lignin. Their existence within the cells is crucial for multiple fundamental processes. The four essential functions of pectin in plant cells include: (a) facilitating adhesion between cells, (b) enhancing the mechanical strength of the cell wall, (c) enabling the formation of stabilising gels, and (d) playing a significant role in the growth of plant cells [31]. 

Many recent research efforts have been devoted to finding high-quality pectin. As a consequence, other components of other plant materials, such as the peels of passion fruit [32,33], pomelo peels [34], peach pomace [35], durian rinds [36,37], mango peels [25], pistachio green hull [38], and sugar beet pulps [39] were thought to include alternative sources of commercial pectin. As mentioned above, in production or commercialisation, the use of these sources is still somewhat restricted. It is possible, especially in developing nations, to find new potential endogenous species and by-products that may be used as sources for additional raw materials to make commercial pectin. The peel of the dragon fruit, a solid waste product from agriculture and industry, is abundant in superior pectin [40].

Pectin is thought to be abundant in dragon fruit peel owing to -1,4-linked D-galacturonic acid (D-galacturonic acid) residues within its heterogeneous structural polysaccharide [41]. Pectin separation from dragon fruit peel is a cutting-edge process since it reduces food waste while increasing the fruit’s monetary worth [42]. 

The extraction conditions for pectin may vary depending on the source from which it is derived. The extraction parameters vary based on the source materials’ characteristics and the process’s economic feasibility. Selecting an appropriate extraction condition is crucial in considering pectin’s chemical properties and is attributed to achieving a high yield while maintaining its quality [43].

#### Pectin Extraction from Dragon Fruit Peels

In essence, the extraction process entails isolating and purifying the pectin that was removed from the source material (plant) [44], resulting in a drying procedure [3]. Extraction parameters, including the temperature of extraction, duration of extraction, pH, and kind of extraction solvents, influence the pectin yield and, ultimately, the pectin quality [3,45,46]. 

Two primary types of extracted pectin can be distinguished based on their degree of esterification: high methoxyl (HM) pectin, which has a degree of esterification ranging from approximately 50 to 80%, and low methoxyl (LM) pectin, which has a degree of esterification below 50%. The formation of various gel types from pectin is contingent upon their esterification level [47]. Factors such as the pH, temperature, time and solvent:liquid (S:L) ratio are usually studied to optimise the extraction conditions [31].

Pectin is often extracted in an industrial environment utilising hot acidified water at a pH of 1.3–3, temperature of 60–100 °C, and times ranging from 20 to 360 min [25]. Nevertheless, prolonged heating can cause pectin to degrade thermally, altering the substance’s physicochemical and functional characteristics and quality [25]. There are many ways to stop this thermal decline, such as burning with microwaves, ultrasonication, and an electromagnetic field with a very high frequency [48]. Bagherian et al. [49] reported that the traditional method exposes companies to acidic environments; thus, researchers have turned to physical methods such as ultrasound and extraction using microwaves instead.

Tongkham et al. [50] found that microwave-assisted extraction enhanced the pectin extraction rate by providing a higher yield in less time than conventional (CV) methods. Their findings demonstrated that extraction rates of pectins from dragon fruit peel rose significantly with microwave treatment and extraction time. This was in line with what was said about extraction from grapefruit with the help of microwaves [49]. Similarly, Rahmati, Abdullah and Kang [51] found that the maximum extraction yielded from the dragon fruit peel under optimal conditions employed at microwave powers of 300, 450, 600 and 800 W were 11.8, 14.9, 17.2 and 18.5%, respectively.

The prevalent methods for obtaining natural pectin from peels include conventional solvent extraction that involves stirring and heating, microwave-assisted extraction (MAE), electromagnetic induction (EMI), ultrasound-assisted extraction (UAE), and enzymatic extraction [31]. Table 4 displays the varieties of extraction methods of natural pectin from peels.

**Figure 1:**
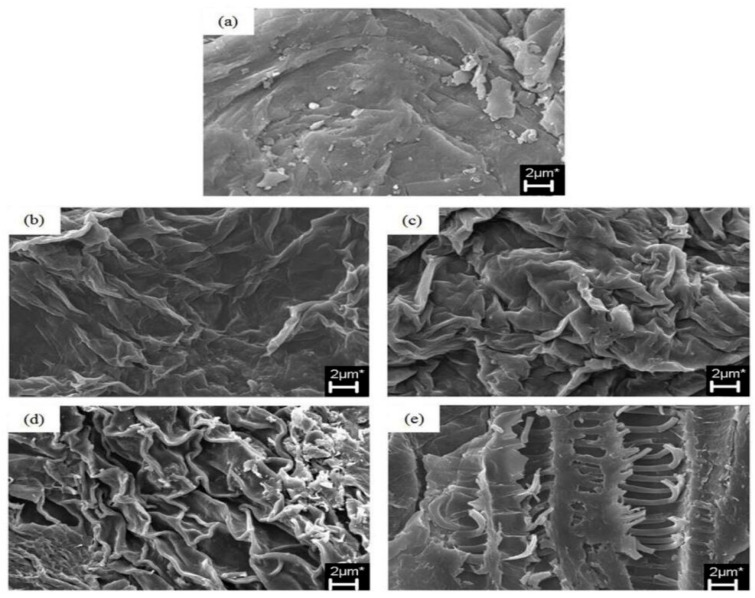
Morphological analysis of dragon fruit peel subjected to microwave powers (**a**) 0 W, (**b**) 300 W, (**c**) 450 W, (**d**) 600 W and (**e**) 800 W [51].

The SEM micrograph of the dragon fruit peel before exposure to microwave radiation (control sample), as shown in Figure 1a, was thoroughly examined by Rahmati et al. [51]. Prior to exposure to microwave radiation, the microscopic structure of the dragon fruit peel remained consistent, compact, and dense. Figure 1b–e displays the SEM images of the microwave-exposed dragon fruit peels. Lower microwave power levels (300 W) caused the microstructure of dragon fruit skins to crease, fold, and deteriorate. The microstructure and interior tissue were harmed and torn when dragon fruit peels were subjected to greater microwave power levels (800 W). The microwave in the experiment can heat the food by penetrating it. Thus, as the microwave power increased, the middle lamella suffered increasing amounts of damage, leading to the complete breakdown and disintegration of the organisation of the primary cell walls of the dragon fruit peels. As a consequence, the extraction yield of pectin increased. Microwave radiation has previously been shown to influence the microstructure of fresh orange peel [55].

### 5.2. Peel Powder and Pectin

The peel must be processed to manage goods with a longer shelf life, as it is a high basis of pectins, phenol, antioxidants, the colour of betacyanin, and total dietary fibres. The peels can be used as well to extract pigments. The extracted pigments can be used for other products to enhance their functional qualities [2].

Table 5 summarises various methods for preparing dragon peel powder (DPP). Spray drying of the peel retained the red colour better than drum drying. Furthermore, because maltodextrin helps to lessen the stickiness of the powder, spray drying the DPP cannot be performed without its prior addition. The use of it may aid in the preservation of betacyanin pigment and other functional qualities [2,56].

**Figure 2:**
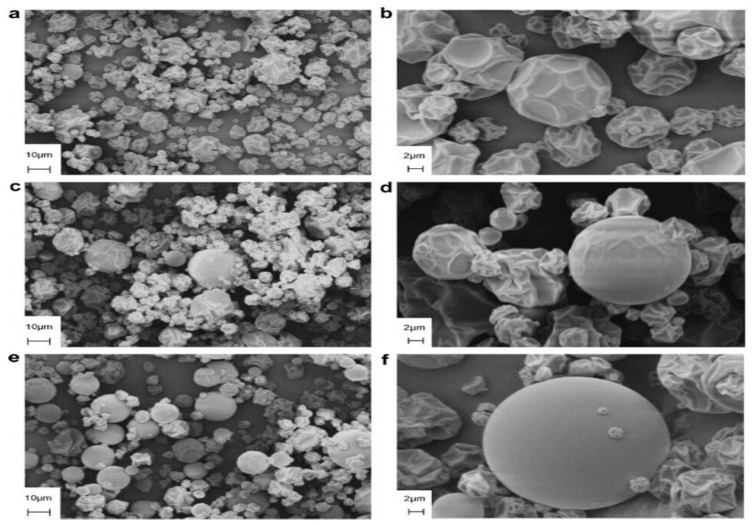
Microstructure of spray-dried dragon fruit peel (DPP) at different temperatures and magnification: (**a**) 138 °C, 2000×; (**b**) 138 °C, 7000×; (**c**) 170 °C, 2000×; (**d**) 170 °C, 7000×; (**e**) 202 °C, 2000×; (**f**) 202 °C, 7000× [60].

A study by Ee et al. [58] showed that the spray-dried DPP contained particles of different sizes in the range of 5–30 μm. A spherical but shrivelled particle might be caused by spray drying taking a long time to dry, as mentioned by Tonon, Brabet and Hubinger [60], who noted that most powder particles remained shortened with shrivelled surfaces, as the inlet air temperature was low, owing to slow dryings. Figure 2 shows the micrographs of spray-dried DPP conducted by Tonon, Brabet and Hubinger at three different temperatures [60].

Researchers have utilised spray and freeze drying to manufacture DPP to preserve their nutritional components, but this procedure has a significant operating cost. Determining the viability of alternative, new, or hybrid drying procedures in terms of cost-effective drying is thus necessary, emphasising small producers and processors [2].

## 6. Current Applications 

### 6.1. Bioplastic from Pectin of Dragon Fruit (Hylocereus polyrhizus) Peel

Food waste or agricultural waste can be potentially used as materials for bioplastics [61]. Most organic waste comes from food sources such as fruit and vegetable scraps, stale meals, and leftovers. Parts such as the peel, seed, stem, and inedible components are generally discarded and not extensively utilised [61,62]. Several authors have considered the potential application of food waste in bioplastics, such as using citrus peels and apple pomace to produce biofilms [63,64,65]. 

The dragon fruit was a subject of study by Listyarini et al. [61] due to its high demand. As it is expected to be developed yearly, the waste can be utilised for bioplastic production. The study began by extracting pectin from the dragon fruit peel. The aim was to make a bioplastic from the pectin of the dragon fruit peel.

The most prominent finding from the analysis was that the pectin yield was a bit low (approximately 11%) compared to a previous study by Zaidel et al. [43], which obtained a pectin yield of 16.20–20.34%. However, no dilute acid was used in the extraction analysis [43], which caused a slight difference in the pectin yield of this study.

Moreover, bioplastics synthesised with and without adding plasticisers exhibited different surface morphologies and behaviours when separated from the printing glass. In this case, ethylene glycol was used as a plasticiser to improve the bioplastic’s elasticity [66]. The results indicated that dragon fruit peel pectins’ bioplastic without ethylene glycol was rigid and challenging to remove from printing glasses compared to the condition with ethylene glycol, which was easier to remove from the printing glass [61]. The presence of ethylene glycol further decreased the density due to the formation of hydrogen bonds in the polymer chain, reducing the stiffness of the bioplastics [67]. 

Another important finding from Listyarini et al. [61] was that the moisture contents of the dragon fruit peel pectins’ bioplastic was 5.71–12%, while pectin and ethylene glycol of the dragon fruit peel were 2.86–5.71%. The FT-IR spectra exhibited that bioplastic belonged in the pectin group, which was revealed by a carbonyl absorption of 1636–1628 cm^−1^ and C-0 by 1098–1101 cm^−1^.

### 6.2. Dragon Fruit Peel (Hylocereus costaricensis) Skin Colour Extract for Bioplastics

The fast development of bioplastics is now solving the issue of non-degradable plastics. Bioplastics are a kind of biodegradable plastic made from biological materials. Although bioplastics can be utilised similarly to conventional plastics, once they have been used up and released into the environment, the final product (which includes water and carbon dioxide gas) will be ruined by the activity of microorganisms. The fact that the raw materials utilised may be updated and are available in large quantities is another benefit of bioplastics. Owing to its biodegradability, availability, eco-friendliness, and cheap cost, starch is one of the most promising and often used constituents in the bioplastics industry [68]. 

Bioplastics can be developed by utilising natural resources such as starch. They are now widely used as packaging for different products. However, two properties affect the quality of bioplastics: physical and mechanical characteristics. Added materials, such as plasticisers, stabilisers, dyes, and antistatics, affect the physical and mechanical characteristics. Putra et al. [69] proposed the extraction of dragon fruit skin (*Hylocereus costaricensis*) to be used as a bioplastic dye since this fruit is easy to find. Most importantly, it is one of the listed fruit wastes. 

The usage of bioplastics in the product’s construction is influenced by its thickness, which is one of the main parameters affecting bioplastic characteristics. The thickness of the bioplastic will impact its gas permeability. The thicker the bioplastic material, the lower the gas permeability and the greater the protection of the packed goods. Other bioplastic mechanical characteristics, such as tensile strength and elongation, may also be affected by thickness. However, bioplastic thickness must be altered to the product it packs when used [69].

### 6.3. Dragon Fruit Peel as a Natural Dye in Food

One of the food’s most crucial qualities is colour, which is widely used as a quality indicator to gauge consumer acceptability. During processing, many naturally coloured foods, including fruit products, are susceptible to colour loss. In order to restore their hues, colourants must be used.

Health-conscious consumers are increasingly compelled to avoid foods containing synthetic colourants. This has prompted the food industry to replace synthetic pigments with natural pigments such as carotenoids, betalains, anthocyanins, and carminic acid. Several studies have demonstrated the possible application of betalains as antioxidant pigments, which motivates their use as a food colouring [70]. 

Betalains can be found in roots, fruits and flowers [71]. Betacyanins, reddish-violet, and betaxanthins, yellowish-orange, are two classes of these water-soluble nitrogen-containing pigments [70]. In addition to cactus fruits such as those of the Opuntia and *Hylocereus genera*, the few known edible sources of betalains include red and yellow beetroot (*Beta vulgaris* L. ssp. *vulgaris*), coloured Swiss chard (*Beta vulgaris* L. ssp. *cicla*), and grain or leafy amaranth (*Amaranthus* sp.) [72,73,74,75]. 

Red beetroot (Beta vulgaris), which includes two main soluble pigments, betanin (red) and vulgaxanthine I (yellow), is the most widely used betalains crop. Betanin makes up the majority of red beets’ betalains spectrum. Hence, colour variation is minimal. Geosmin and certain pyrazines also have an unfavourable earthy taste, which makes them unattractive for usage, for example, in dairy products [74,76].

As a result, efforts have been undertaken to investigate other sources of betalains. The Cactaceae family of betalain-producing plants is the most promising. Among these, pitayas (*genera Cereus*, *Hylocereus*, *and Selenicereus*) and cactus pears (*genus Opuntia*) are the most widely grown fruit crops, which are most suited for research as a source of betalain for food-colouring goods [70,77,78].

In contrast to beetroot extracts, cactus fruits can be used in food without adverse flavour effects. Cactus fruits contain betalains that exhibit a more comprehensive range of colours, ranging from yellow–orange in *Opuntia* sp. to red–violet in *Hylocereus* sp., in contrast to red beetroot. This may open new windows of colour diversification [70]. The fact that cactus fruits need little water and soil is another benefit. They are seen as different cultures for agricultural economies in arid and semiarid areas [79]. 

Dragon fruit peel can be employed as a raw material for pigment extraction due to its betalain content, presenting attractive and stable colours [80]. It contains phenolic compound betalains [81], a water-soluble pigment consisting of two structural groups [82]. 

Water was used in an effective extraction procedure to preserve the stability of delicate pigments such as betacyanin [83]. This resulted in better pigment stabilisation [70]. Delgado-Vargas, Jimenez and Lopez [84] stated that in most cases, ethanol or methanol solution (20–50%) must be used to finish extraction. Slight acidification of the extraction medium enhances betacyanin stability and avoids oxidation by polyphenoloxidases [71,85].

### 6.4. Dragon Fruit Peel as a Raw Material in the Cosmetic Industry

In light of rising awareness among consumers of the long-term detrimental consequences of synthetic chemicals, natural cosmetics have become a big trend in recent years. Lip care products are among the various types of cosmetics available on the market since they are so often utilised [86]. Afandi et al. [86] stated that the oil–wax basis of a lipstick is what gives it its characteristic stickiness. Oil is used to dissolve or disperse a staining dye, which is then flavoured, shaped, and packaged. Nonetheless, the present demand for lip care products, however, emphasises both the medical benefit that is better for lips and the cosmetic value. As a result, the market has seen the emergence of medicated lipsticks containing active pharmaceutical components [86], in addition to giving moisture and emollient actions to inhibit cracking chapped lips. Aher et al. [87] claimed that medicated lipstick might grant a shield against bacterial infection, owing to the active components in the formulation. Many scientists are interested in the Cactaceae member *Hylocereus Polyrhizus*, or dragon fruit, because of its exotic flavour, unusual appearance, and potential as a natural food dye [86]. 

## 7. Summary 

Dragon fruit (*Hylocereus polyrhizus*), also known as pitaya, is a Cactaceae family member with two distinct genera: ‘*Hylocereus*’ and ‘*Selenicereus*’. The *Hylocereus* genus, which has roughly 16 different species, contains the most often farmed types. Each part of the dragon fruit has high levels of antioxidants, fibre, vitamin C, and minerals (mainly calcium and phosphorus). Researchers have paid close attention to the fruit because of its nutritional properties. It may be converted into various items, such as pectin extract for bioplastics, colour extract for bioplastics, natural dye and materials for cosmetics. 

The pitaya peel accounts for approximately 22% of the total pitaya fruit and is currently being discarded. The substance exhibits many pectins, betacyanin pigment, and overall dietary fibre. The ratio of insoluble dietary fibre (IDF) to soluble dietary fibre (SDF) in the peel is favourable, measuring at 3.8:1.0. Therefore, the utilisation of pitaya peel can serve as a valuable source of dietary fibre, pectin, and natural pigmentation.

There are a few methods available to prepare dragon fruit peels into peel powder. Different methods will contribute to various advantages and disadvantages in retaining the nutritional composition of the fruit peels. The most commonly used were drum drying, spray drying and tray drying. The functional qualities and operating costs are the things to consider for manufacturing dragon fruit peels. 

Because of its widespread popularity, additional studies are required to fully comprehend the properties of the dragon fruit and its constituents. Although several kinds of research related to this issue have been conducted for some applications, there is still significant room for its varied and matured development in other research areas. 

## Figures and Tables

**Table 1:** Dragon fruit can be processed into a variety of goods.

Dragon fruit	Pulp	Fresh cut
		Juice
		Juice powder
		Jelly
		Jam
	Peel	Peel powder
		Pectin
		Colour extract
	Seed	Seed oil
		Tree seedling

**Table 2:** Chemical properties of the pulp of different types of S. queretaroensisa, S. griseus, S. stellatus, and S. pruinosus [15].

No.	Variety	pH	Acidity (Malic Acid and Citric Acid %)	Total Sugar (%)
1	Amarilla	3.90	0.50	11.0
2	Blanca	4.70	0.18	11.0
3	Mamey	5.00	0.15	10.0
4	Morada	4.60	0.29	11.0
5	Roja	4.90	0.17	10.0
6	Ahuatlan municipality	4.96	0.11	8.8
7	Santa Clara Huitziltepec municipality	4.97	0.09	8.9
8	*S. griseus*	5.20	0.12	12.2
9	Roja	4.20	0.53	9.2
10	Blanco	4.40	0.39	10.0
11	Amarillo	4.46	0.50	9.1
12	Solferina	3.70	0.47	9.0
13	*S. stellatus*	3.95	0.64	9.1
14	Red	-	0.17	9.3
15	Orange		0.13	10.3

**Table 3:** Carbohydrate components of dragon fruit (Hylocereus polyrhizus) [7].

Carbohydrates Compositions	Percentage (%)
Cellulose	9.25 ± 1.33
Starch	11.07 ± 0.03
Pectin	10.79 ± 0.01
Lignin	37.18 ± 1.02
Sugars i. Glucose ii. Maltose iii. Fructose iv. Sucrose v. Galactose *Total sugars*	4.15 ± 0.03 3.37 ± 0.01 0.86 ± 0.02 ND ND 8.38
Total dietary fibre i. Insoluble ii. Soluble iii. Ratio of IDF SDF	69.30 ± 0.53 56.50 ± 0.20 14.82 ± 0.42 3.8:1.0

The values are means ± standard error (n = 3), ND: not detected.

**Table 4:** Varieties of high added-value dragon fruit peel pectin extraction methods.

Extraction Method	Model	Parameters	Pectin Yield Extraction (%)	Ref.
Microwave-Assisted Extraction (MAE)	Respond surface methodology (RSM)	pH 2.07, 65 s and solid–liquid ratio of 66.57 g/mL	18.53	Rahmati et al. [42]
	Respond surface methodology (RSM)	450 W, 5 min	21.68	Tongkham et al. [50]
	Respond surface methodology (RSM)	400 W, 45 °C, 20 min, and solid–liquid ratio of 24 g/mL	7.5	Thirugnanasambandham, Sivakumar and Prakash Maran [52]
Ultrasound-Assisted Extraction (UAE)	Respond surface methodology (RSM)	71.8 °C, 25 min, and solid–liquid ratio of 35.6 g/mL	7.49	Lin, Kai and Ali [53]
	-	45 °C, 30 min	9.38	Nguyen and Pirak [54]

**Table 5:** Varieties of drying methods for peel powder preparation.

Drying Method	Varieties Used	Operational Parameters	Findings	Ref.
Drum Drying	Red dragon fruit (*Hylocereus polyrhizus*)	Drum speed: 1 rpm Pressure of steam: 2 bar Gap of drum: 0.1 mm	Water contents: 10.66% w.b. Activity of water: 0.420 Betacyanin contents: 80.21 mg/g of dm Water holding capacity: 2.523 g water/g sample 3.328 mg trolox/g dm of decrease in radical scavenging activity Swelling capacity: 6.23 mL/g Solubility: 51.44% 98.62% total phenolic content retention Oil holding capacity OHC: 3.57 g/g Powder density: 0.1315 g/mL	[8]
Spray Drying	Red dragon (*Hylocereus polyrhizus*) and white dragon (*Hylocereus undatus*) fruit	Inlet temperature: 120–180 °C (red) and 110–180 °C (white) Outlet temperature: 95 °C Concentration of maltodextrin DE 10: 30, 35, 40, 45, 50% (*w*/*w*)	The highest yield of spray-dried red and white dragon fruit: at inlet air temperatures of 120 and 110 °C, respectively, and threshold maltodextrin concentration of 30% (*w*/*v*)	[57]
Spray Drying	Red pitaya fruits (*Hylocereus* *polyrhizus*)	Inlet temperatures: 149, 155, 165, 175, and 181 °C Outlet air temperatures: 72, 75, 80, 85, and 88 °C Concentration of maltodextrin DE 10: 4, 8, 15, 22, and 26% (*w*/*w*)	Optimised condition attained: 165 °C of inlet temperatures, 80 °C of outlet air temperatures and 15% (*w*/*w*) of maltodextrin DE 10 Moisture content: 3.30% w.b. Water activity: 0.30 Betacyanin retention: 87.62% Solubility: 93.03% Hygroscopicity: 28.21%	[56]
Spray Drying	Red pitaya fruit (*Hylocereus* *polyrhizus*)	Inlet temperatures: 165 °C Outlet air temperatures: 80 °C Concentrations of maltodextrin DE 10: 15% (*w*/*w*) Powder was stored in low-density polyethylene bags for 14 weeks at accelerated (45 2 °C; 38% RH) and 6 months at room temperature (26 2 °C, 50–70% RH).	Water contents: 3.30% Water activities: 0.299 Betacyanin content: 64.66 mg/100 g Hygroscopicity: 27.63% Solubility: 89.83% At accelerated temperature, the powder’s half-life was estimated to be about 76.2 weeks, and at normal temperature, 38.3 months At both storage temperatures, the betacyanin pigment was retained by more than 86%.	[58]
Tray Drying	White dragon fruit (*Hylocereus undatus*)	Peel of 6 × 2 cm strips Forced air temperature: 50, 60, 70 °C Air speed: 1.0 m/s	Increase in the drying temperature from 50 to 70 °C led to increases in: pH: 5.06–5.13 ascorbic acid: 11.38–16.11 mg/100 g and decreases in: moisture content: 5.39–4.40% w.b. water activities: 0.353–0.318 betacyanins: 51.81–17.25 mg/100 g betaxanthins: 63.50–35.22 mg/100 g Optimum drying temperature: 50 °C	[59]

## Data Availability

Data presented in this study are available on request from the corresponding author.
